# Prevalence of Optic Disc Hemorrhages in Rural Central India. The Central India Eye and Medical Study

**DOI:** 10.1371/journal.pone.0076154

**Published:** 2013-09-30

**Authors:** Jost B. Jonas, Vinay Nangia, Anshu Khare, Maithili Kulkarni, Arshia Matin, Ajit Sinha, Krishna Bhojwani, Prabhat Nangia, Songhomitra Panda-Jonas

**Affiliations:** 1 Suraj Eye Institute, Nagpur, India; 2 Department of Ophthalmology, Medical Faculty Mannheim of the Ruprecht-Karls-University, Heidelberg, Mannheim, Germany; Oregon Health & Science University, United States of America

## Abstract

**Purpose:**

To determine the frequency of optic disc hemorrhages in a rural Indian population.

**Methods:**

The population-based Central Indian Eye and Medical Study included 4711 subjects. Mean age was 48.5±12.9 years (range: 30-100 years). Color optic disc photographs were examined.

**Results:**

Optic disc photographs were available for 4570 (97.0%) subjects. Prevalence of disc hemorrhages was 17/8869 (0.19%; 95%CI:0.10,0.28) per eye and 16/4570 (0.35±0.09%; 95%CI:0.18,0.52) per subject. Prevalence of disc hemorrhages increased from 0.05% (95%CI:0.00,0.13) in the age group of 30-39 years to 0.25% (95CI:0.00,0.49) in the age group of 60-69 years and to 0.91% (95%CI:0.24,1.58) in the age group of 70+ years. After adjusting for older age, higher systolic blood pressure, diabetes mellitus, myopic refractive error, smaller neuroretinal rim area and thinner retinal nerve fiber layer, occurrence of disc hemorrhages was associated only with glaucomatous optic nerve damage (*P*<0.001; Odds ratio: 87; 95%CI:32,239). Eleven of the 17 (65%; 95%CI:39,90) disc hemorrhages were found in glaucomatous eyes. Out of 193 glaucomatous eyes, 11 eyes (5.7%; 95%CI:2.4,9.0) showed a disc hemorrhage. Out of the 8676 non-glaucomatous eyes, 6 eyes (0.07%; 95%CI:0.01,0.12) had an optic disc hemorrhage.

**Conclusions:**

Prevalence of disc hemorrhages (0.2% per eye; 0.4% per subject) in Indians aged 30+ years was strongly associated with glaucoma after adjustment for age, blood pressure and diabetes mellitus. A disc hemorrhage suggested glaucomatous optic nerve damage with a positive predictive value of 65%. About 6% of glaucomatous eyes showed a disc hemorrhage at the time of clinical examination highlighting the importance of optic disc hemorrhages for the diagnosis of glaucoma.

## Introduction

Hemorrhages at the border or inside of the optic nerve head have been considered to be a hallmark for glaucomatous optic neuropathy [1-9]. Optic disc hemorrhages play an important role in the ophthalmoscopic diagnosis of optic nerve diseases. Consequently, numerous hospital-based studies and several population-based studies have examined the prevalence and locations of disc hemorrhages in various ethnic groups and in various groups of patients with glaucoma [1-10]. Hospital-based studies have the major disadvantage of a potential selection artifact due to a referral bias by the referring ophthalmologists. The population-based studies which examined disc hemorrhages included study participants from Western countries or from regions such as Beijing in China where a developed infrastructure was present and where the lifestyle of the general population had been mostly adapted to an international level [10-20]. These population-based investigations included subjects with an age of 40+ years or 49+ years. Since none of the previous studies was performed on a population which lived in an environment with a relatively low degree of development, and since younger subjects were usually not included in the previous investigations, we conducted a population-based study in a remote area in rural Central India where many aspects of the living conditions had not markedly changed during the last century and in which we included subjects with an age of 30 years onwards.

## Methods

### Ethics Statement

The Medical Ethics Committee of the Medical Faculty Mannheim of the Ruprecht-Karls-University Heidelberg and the ethical committee of Suraj Eye Institute / Nagpur approved the study and all participants gave informed written consent.

The Central India Eye and Medical Study (CIEMS) is a population-based cross-sectional study performed in rural Central India in 8 villages in a distance of 40 km to the next city (Nagpur) [21]. The villages were surrounded by so called tribal areas. Out of the 5885 eligible subjects, 4711 (80.1%) subjects (2191 men (46.5%)) participated. The mean age was 49.5 ± 13.4 years (range: 30-100 years), the mean reported monthly income was 1584 ± 1233 rupees (one US dollar equals roughly fifty rupees), and the rate of self-reported illiteracy was 35%. The group of study participants and the group of non-participants (n=1174) did not differ significantly in age (49.5 ± 13.4 years versus 48.6 ± 14.1 years; *P*=0.06); the proportion of men was significantly (*P*<0.001) higher in the group of non-participants (46.5% versus 58.3%).

A hospital bus brought the study participants in the morning from the villages to the hospital, where a series of examinations was performed during the whole day. Trained social workers filled out a questionnaire for the participants; this questionnaire included questions regarding socioeconomic background and living conditions, tobacco use and alcohol consumption, and any known diagnosis of major systemic diseases. Pulse, arterial blood pressure, and body height and weight were recorded. One-and-a-half hours after a standardized lunch, blood and urine samples were obtained and biochemically analyzed. Diabetes was defined as a postprandial blood glucose concentration ≥11.2 mmol/L (200 mg/dL), a blood concentration of glycosylated hemoglobin (Hb1Ac) of ≥6%, or a self-reported medical diagnosis of diabetes (any prior diagnosis of diabetes by a health care professional). Arterial hypertension was defined as a systolic blood pressure ≥140 mm Hg and/or a diastolic blood pressure ≥90 mm Hg, and/or self-reported current treatment for arterial hypertension with antihypertensive medication.

Uncorrected and best corrected visual acuity was measured. Visual field examinations were performed with frequency-doubling perimetry using the screening program C-20-1 (Zeiss-Humphrey, Dublin, CA). Intraocular pressure was measured by applanation tonometry. Slit lamp biomicroscopy was carried out by a fellowship-trained ophthalmologist, and any abnormality of the anterior segment was noted. Corneal pachymetry and ocular biometry were performed by sonography using the Pacscan (Sonomed, Lake Success, NY). Using the slit lamp, photographs of the limbal region were taken to assess the limbal anterior chamber depth at the most peripheral part of the cornea. Gonioscopy was performed for all study participants in dim illumination using the magnaview single mirror goniolens (Ocular Instruments, Bellevue, WA, USA). In subjects with any extent of occludable angles, indentation gonioscopy was performed with the Sussman 4 mirror goniolens (Ocular Instruments, Bellevue, WA, USA). The pupil was dilated using tropicamide 0.8% and phenylephrine 5% three times at 15 minute intervals so that all subjects attained maximal pupillary dilatation. A second slit lamp examination was performed to assess the presence of pseudoexfoliation. Digital photographs of the lens were taken. Digital photographs of the optic disc (20 degrees; fundus camera type CR6-45NM, Canon Inc. U.S.A.) and macula (50 degrees; Zeiss FF450 telecentric fundus camera (Zeiss, Meditec Co., Oberkochen, Germany)) were obtained. Magnification by optic media was corrected for by a built-in algorithm and we measured the area and horizontal diameter and vertical diameter of the optic disc and cup.

Using the optic disc photographs, we assessed the prevalence of optic disc hemorrhages. An optic disc hemorrhage was defined as a hemorrhage touching the optic disc border or lying completely inside of the optic nerve head, which was considered as all area within the peripapillary ring^6^. Glaucoma was defined by the criteria of the International Society of Geographical and Epidemiological Ophthalmology (ISGEO) classification scheme [22]. In a second step, glaucoma was diagnosed based on a glaucomatous appearance of the optic disc. The optic nerve head was glaucomatous (1) if the inferior-superior-nasal-temporal (ISNT)-rule of the neuroretinal rim shape was not fulfilled in early glaucoma and in eyes with a normally shaped optic disc (it included a notch in the neuroretinal rim in the temporal inferior region and / or the temporal superior region); or (2) if an abnormally large cup was present in a small optic disc which normally would not show cupping. The assessment of the optic disc photographs was carried in a masked manner without knowledge of intraocular pressure or the perimetric results. Each photograph of a glaucomatous optic disc was independently adjudicated by two senior graders (VN and JBJ). In addition to the optic disc photographs, confocal laser scanning tomograms (HRT, Heidelberg Engineering, Heidelberg, Germany) of the optic disc were taken for all eyes. The whole glaucoma group was differentiated into subjects with open-angle glaucoma and with primary angle closure glaucoma. Open-angle glaucoma was characterized by an open anterior chamber angle, in addition to a normal depth of the anterior chamber as assessed by slit lamp biomicroscopy. In angle-closure glaucoma, the anterior chamber angle was occluded or occludable. The anterior chamber angle was defined as occludable, if more than 270° of the posterior trabecular meshwork could not be seen upon gonioscopy [22-24].

Only those subjects with evaluable optic disc photographs were included into the study. Statistical analysis was performed using a commercially available statistical software package (SPSS for Windows, version 20.0, IBM-SPSS, Chicago, IL). In a first step, we determined the prevalence of disc hemorrhages (presented as mean ± standard error). In a second step, we performed univariate analyses of the associations between the presence of disc hemorrhages and other ocular and systemic parameters. In the third step, we carried out binary regression analyses with the presence of disc hemorrhages as the dependent parameter and all parameters as independent variables which were associated significantly with the presence of disc hemorrhages in the univariate analyses. We then removed step by step all independent parameters for which the *P*-values were higher than 0.05, starting with the parameters with the highest *P*-values, until all remaining independent parameters were significantly associated with the presence of disc bleedings. All *P*-values were two-sided. Odds ratios (OR) and 95% confidence intervals (CI) were presented.

## Results

Photographs of the optic nerve head disc were available for 8869 (94.1%) eyes of 4570 (97.0%) subjects. The mean age of the 4570 participants (53.8% women) was 48.5 ± 12.9 years (median: 45.0 years; range: 30-100 years). Age groups from 30-39 years, 40-49 years, 50-59 years, 60-69 years, and 70+ years, included 23.8%, 29.2%, 17.1%, 19.1% and 11.0% of the study population respectively. The mean axial length was 22.64 ± 0.86 mm (median: 22.61 mm; range: 18.08 to 32.70 mm), and the mean refractive error was -0.06 ± 1.70 diopters (median: +0.12 diopters; range: -22.0 to +9.38 diopters). The group of subjects with assessable disc photographs versus the group of subjects without optic disc assessment was significantly younger (48.5 ± 12.9 years versus 64.6 ± 12.9 years; *P*<0.001), was significantly less myopic (-0.06 ± 1.70 diopters versus -1.05 ± 2.54 diopters; *P*<0.001), had significantly shorter axial length (22.64 ± 0.86 mm versus 22.87 ± 1.32 mm; *P*<0.001), and had significantly fewer women (49.0% versus 53.8%; *P*=0.03). Intraocular pressure did not vary significantly between both groups (13.8 ± 3.4mm Hg versus 13.8 ± 4.6 mmHg; *P*=0.78).

Optic disc hemorrhages were detected on 17/8869 optic disc photographs (16 subjects) indicating a prevalence of 0.19 ± 0.05% (95% confidence interval: 0.10, 0.28) per eye and a prevalence of 0.35 ± 0.09% (95%CI: 0.18, 0.52) per subject.

In univariate analysis, presence of optic disc hemorrhages was significantly (*P*<0.05) associated with older age, higher systolic blood pressure, presence of arterial hypertension and diabetes mellitus, myopic refractive error, neuroretinal rim area, retinal nerve fiber layer cross sectional area, and presence of glaucomatous optic nerve damage (Table 1). Prevalence of disc hemorrhages increased from 0.05% (95%CI: 0.00, 0.13) in the age group of 30-39 years, 0.11% (95%CI: 0.00, 0.24) in the age group of 40-49 years and 0.13% (95%CI: 0.00, 0.30) in the age group of 50-59 years to 0.25% (95%CI: 0.00, 0.49) in the age group of 60-69 years and to 0.91% (95%CI:0.24,1.58) in the age group of 70+ years. The occurrence of optic disc hemorrhages was statistically not significantly associated with optic disc area (*P*=0.68) (Table 1).

**Table 1:** Differences between Eyes with Optic Disc Hemorrhages and Eyes without Optic Disc Hemorrhages in the Central India Eye and Medical Study.

	Hemorrhage Group	Non-Hemorrhagic Group	*P*-Value
n (Eyes)	17	8852	
Age (Years)	60.8 ± 13.5	48.5 ± 12.8	<0.001
Systolic Blood pressure (mmHg)	143 ± 25	124.2 ± 20.7	0.008
Diastolic Blood pressure (mmHg)	78 ± 13	74.5 ± 11.6	0.32
Arterial Hypertension (%)	47.1 ± 12.5	20.5 ± 0.05	0.01
Blood Concentration of:			
Glucose (mg/dL)	120.6 ± 47.5	109.3 ± 27.9	0.35
Glycosylated Hemoglobin (%)	5.27 ±0.93	4.63 ± 1.78	0.12
High-Density Lipoproteins (mg/dL)	42.6 ± 36.4	35.0 ± 58.5	0.42
Cholesterol (mg/dL)	4.28 ± 1.34	4.56 ± 0.73	0.30
Diabetes Mellitus (%)	28.6 ± 18.4	5.5 ± 0.3	0.05
Refract. Error (Diopters)	-1.23 ± 2.95	-0.07 ± 2.68	0.002
Axial Length (mm)	22.2 ± 1.6	22.6 ± 0.9	0.30.
Central Corneal Thickness (µm)	501 ± 37	514 ± 33	0.21
Anterior Chamber Depth (mm)	3.50 ± 0.67	3.23 ± 0.35	0.16
Disc area (mm^2^) Zeiss	2.74 ± 0.53	2.79 ± 0.80	0.68
Disc area (mm^2^) HRT	2.06 ± 0.75	2.19 ± 0.46	0.50
Neuroretinal Rim Area (mm^2^) Zeiss	1.23 ± 0.48	1.63 ± 0.34	0.13
Neuroretinal Rim Area (mm^2^) HRT	1.59 ± 0.48	1.78 ± 0.52	0.008
Retinal Nerve Fiber layer Cross Section Area	0.90 ± 0.59	1.30 ± 0.39	0.02
Intraocular Pressure (mmHg)	15.6 ± 7.9	13.8 ± 3.4	0.55
Prevalence of:			
Glaucomatous Optic Nerve Damage (%)	11/17 (65%)	182/8852 (2.1%)	<0.001
Open-Angle Glaucoma (%)	9/17 (53%)	164/8852 (1.9%)	<0.001
Angle-Closure Glaucoma (%)	2/17 (12%)	18/8852 (0.2%)	0.001
Diabetic Retinopathy (%)	0.00	0.2 ± 0.1	1.00
Retinal Vein Occlusion (%)	0.00	0.4 ± 0.1	1.00
Myopic Retinopathy (%)	0.00	0.3 ± 0.1	1.00

The data are given as mean ± standard deviation; frequencies are described mean ± standard error; *P*-Value: statistical significance of the difference between the two study populations

We then performed a binary logistic regression analysis with the presence of disc hemorrhages as dependent variable and all variables as independent parameters which showed a significant association (*P*<0.05) with the presence of disc hemorrhages in the univariate analysis. After step-wise dropping those independent parameters which were no longer significantly associated with disc hemorrhages (prevalence of diabetes mellitus (*P*=1.00), refractive error (*P*=0.77), age (*P*=0.99), retinal nerve fiber layer cross section area (*P*=0.49), systolic blood pressure (*P*=0.15) and neuroretinal rim area (*P*=0.09)), only presence of glaucomatous optic nerve damage remained to be significantly associated with the presence of a disc hemorrhage (OR: 87; 95%CI: 32, 239; *P*<0.001).

A similar result was obtained if the ISGEO definition of glaucoma was applied after which the presence of disc hemorrhages was significantly associated with the presence of glaucoma (OR: 14.5; 95%CI: 4.0, 51.9; *P*<0.001) and older age (OR: 1.07; 95%CI: 1-03, 1.11; *P*=0.001).

Eleven of the 17 (11/17 or 65% (95%CI: 39, 90)) disc hemorrhages were found in glaucomatous eyes. Out of these 193 glaucomatous eyes, 11 (11/193 or 5.7% (95%CI: 2.4, 9.0)) showed a disc hemorrhage. Out of the 8676 non-glaucomatous eyes, 6 (6/8676 or 0.07% (95%CI: 0.01, 0.12)) eyes showed an optic disc hemorrhage. Axial length in these 6 eyes ranged between 22.02 mm and 23.27 mm, and best corrected visual acuity ranged between 1.0 (n=1), 0.25 (n=1), 0.20 (n=1), 0.16 (n=2) and hand movements (n=1). Reasons for the visual impairment in 5 eyes were advanced cataract (n=4) and age-related macular degeneration (n=1) with detected association to the optic disc hemorrhage.

Two (1.6%) eyes out of 122 eyes with an intraocular pressure higher than 21 mmHg showed a disc hemorrhage, with the 122 eyes with elevated intraocular making out 1.4% of the whole study population. Correspondingly, the prevalence of disc hemorrhages was not significantly correlated with intraocular pressure (*P*=0.55) (Table 1). Two (12%) out of the 17 disc hemorrhages were detected in eyes with an elevated intraocular pressure. The two eyes with an elevated intraocular pressure had glaucomatous optic nerve damage. Within the glaucoma group, the prevalence of disc hemorrhages was not significantly associated (*P*=0.33) with neuroretinal rim area.

Dividing the glaucomatous group into a normotensive subgroup with the single intraocular pressure measurement ≤21 mm Hg and a hypertensive subgroup with the single intraocular pressure measurement higher than 21 mm Hg showed that the frequency of optic disc hemorrhages did not vary significantly (5.66 ± 1.84% versus 5.88 ± 4.10%; *P*=1.00) between the two subgroups. Dividing the glaucoma group into eyes with open-angle glaucoma (n=173) and angle-closure glaucoma (n=20) showed that the prevalence of disc hemorrhages did not vary significantly between both groups (5.20 ± 1.69% versus 10.0 ± 6.88%; *P*=0.32).

## Discussion

In our population-based study in rural Central India, the prevalence of splinter-shaped or flame-shaped hemorrhages at the optic disc was 0.19 ± 0.05% or 17 out of 8869 eyes. The main factor associated with disc hemorrhages was the presence of glaucomatous optic neuropathy, independently of intraocular pressure.

The frequency of disc hemorrhage found in our study was lower than the figure from other population-based studies such as the Blue Mountains Eye Study (prevalence of optic disc hemorrhage: 1.4%) [11], the Beaver Dam Study (0.93) [10], the Japanese Tajimi study (0.3%) [18], and The Beijing Eye Study (1.24%) [20]. The reasons for the discrepancy between the studies may have been differences in the inclusion criterion of age (Blue Mountains Eye study: 49+ years; Beaver Dam Study: 43-84 years; Tajimi Study: 40+ years; Beijing Eye Study: 40+ years; our study: 30+ years) and in the living conditions of the study population, with very rural basic conditions in our study and medium to highly developed conditions in the other studies. To cite an example, the very basic living conditions in our study population may have been of the reasons why the prevalence of diabetes mellitus decreased beyond an age of 65 years in our study potentially due to a higher mortality of patients with diabetes mellitus [25]. Correspondingly, the rate of diabetic retinopathy in the diabetic subgroup of our study population was markedly lower (5.5% versus about 30%) than in studies from more developed regions [26]. Although a disc hemorrhage may not be a risk factor for an increased mortality, the differences in the living conditions may serve to partially explain differences in the results between our study and investigations on other ethnic groups.

In all studies, the main factor associated with disc hemorrhages was glaucoma [1-20]. As in previous studies, the prevalence of disc hemorrhages was not significantly associated with intraocular pressure and did not vary significantly between glaucomatous with a single normal intraocular pressure and glaucomatous with a single elevated intraocular pressure [20]. Correspondingly, eyes with open-angle glaucoma and eyes with angle-closure glaucoma did not vary significantly in the prevalence of disc hemorrhages. It is in contrast to the observations made in the Blue Mountains Eye Study in which the prevalence of disc hemorrhages was 8% in open-angle glaucoma subjects with high-pressure glaucoma and 25% in low-pressure glaucoma [11].

Within the glaucoma group, 5.7% (95%CI: 2.4, 9.0) of the eyes showed a disc hemorrhage. This figure is roughly comparable to, or slightly lower than, figures from previous population-based studies. The prevalence of disc hemorrhages in the glaucoma patients was 8.2% in the Tajimi study [18], 13.8% in the Blue Mountain Study [11], and 8.8% (95%CI: 5.12%, 12.58%) in The Beijing Eye Study [20]. Correspondingly, hospital-based studies revealed a prevalence of disc hemorrhage in patients with glaucoma of about 4% to 7% [6]. As a corollary, 0.07% (95%CI: 0.01, 0.12) of the non-glaucomatous eyes showed an optic disc hemorrhage in our study, a figure which again was comparable to, or slightly lower than, figures from other studies such as the Tajimi study (0.2%) [18].

From a clinical point of view, the results suggest that the positive predictive value of an optic disc hemorrhage for glaucoma was about 65% since 65% of the observed optic disc hemorrhages were found in glaucomatous eyes. Accordingly, the odds ratio of the association between an optic disc hemorrhage and glaucoma was high (OR: 87). These figures were higher than in previous population-based studies such as The Beijing Eye Study in which 19% of the disc hemorrhages were found in glaucomatous eyes [20]. Hospital-based studies had underlined the importance of disc hemorrhages for the diagnosis of glaucoma, since disc hemorrhages had been reported only rarely for non-glaucomatous eyes in hospital-based studies [6]. Previous population-based studies, such as the Beaver Dam Study and the Blue Mountains Eye Study, had challenged that assumption, since a considerable number of disc hemorrhage was found in non-glaucomatous eyes [10,11,20]. In the present investigation, the specificity of disc hemorrhages for glaucoma was higher than in the previous other population-based studies, but lower than in the hospital-based investigations.

Possible limitations of the present investigation should be considered. First, selection bias could have accentuated some estimates and masked others. The overall participation rate in our survey was 80.1%, and it is possible that non-participants had different rates of disc hemorrhages. Second, the subjects without assessable disc photographs were significantly older than the subjects with disc photographs. Since the prevalence of disc hemorrhages strongly depended on older age, one may assume that the prevalence of disc hemorrhage in our study was under-estimated. Third, the number of eyes with disc hemorrhages was relatively low, so that a statistically meaningful subgroup analysis was not possible. Fourth, an optical coherence tomography of the vitreo-retinal surface was not performed, so that we could not assess the association between a posterior vitreous detachment and disc hemorrhages [27].

In conclusion, optic disc hemorrhages occurred in a frequency of about 0.2% per eye and 0.4% per subject in Indians aged 30+ years. The main factor associated with optic disc hemorrhages was glaucomatous optic neuropathy after adjustment for age, blood pressure and diabetes mellitus. Presence of a disc hemorrhage suggested glaucomatous optic nerve damage with a positive predictive value of about 65%. About 6% of glaucomatous eyes showed a disc hemorrhage at the time of examination in the present study highlighting the diagnostic importance of optic disc hemorrhages for the diagnosis of glaucoma.
